# Meynert and the basal nucleus

**DOI:** 10.1590/S1980-57642013DN74000013

**Published:** 2013

**Authors:** Eliasz Engelhardt

**Affiliations:** 1 Neurologist, Full Professor (retired), Cognitive and Behavioral Neurology Unit –INDC-CDA/IPUB − Federal University of Rio de Janeiro (UFRJ), Rio de Janeiro-RJ, Brazil.

**Keywords:** history, Reil, Gratiolet, Meynert, Koelliker, *Substantia innominata*, *Ansa peduncularis*, *nucleus basalis*, basal nucleus, cholinergic

## Abstract

Meynert described the "loop of the peduncular foot" *(Schlinge des Hirnschenkelfusses),* and its ganglion *(Ganglion der Hirnschenkelschlinge)* and related them to Reil's *Substantia innominata* and Gratiolet's *Ansa peduncularis,* from which he apparently built up his findings. Koelliker renamed the ganglion with the eponymous designation *Meynert'sches Basalganglion* (Meynert's basal ganglion), a name which endures to the present day, and described its topographical spread in relation to neighboring structures. Meynert and Koelliker also described aspects of cell composition of the ganglion (or nucleus) with a better account of the latter. Both, together with Reil and Gratiolet, were the outstanding personalities of the 19^th^ century who performed the pioneering studies on basal formations of the forebrain. After these works, a considerable body of research appeared in the 20^th^ century, with a focus on Meynert's basal nucleus and related structures. The development of further knowledge about these structures revealed their great importance in the activity of the brain, as evidenced in both normal and pathological states.

## Abstract (pt)

Meynert descreveu a "alça do pé do pedúnculo" *(Schlingedes Hirnschenkelfusses)* e seu gânglio *(Ganglion der Hirnschenkelschlinge),* relacionando-os à *Substantia innominata* de Reil e à *Ansa peduncularis* de Gratiolet, a partir dos quais aparentemente desenvolveu seus achados. Koelliker renomeou o gânglio com a designação epônima de *Meynert'sches Basalganglion* (gânglio basal de Meynert), que perdura até o presente, e descreveu sua extensão topográfica em relação às estruturas vizinhas. Meynert e Koelliker descreveram também aspectos da composição celular do gânglio (ou núcleo), com um relato melhor do segundo. Ambos, juntamente com Reil e Gratiolet, foram as personalidades de destaque do século 19 que realizaram os estudos pioneiros sobre formações basais do prosencéfalo. Após esses, um número considerável de estudos apareceu no século 20, com foco no núcleo basal de Meynert e estruturas relacionadas. O desenvolvimento ulterior do saber sobre as mesmas mostraram sua grande importância na atividade cerebral, como visto em condições normais e patológicas.

## INTRODUCTION

As for almost all knowledge in neuroscience, a long period elapses, sometimes a century or more, in the transition from anatomical research to the resulting corollary application. This is the case for the basal structures of the forebrain, and the included cholinergic nuclei, which involved some of the most outstanding personalities of the 19^th^ and 20^th^ centuries. The highlights of this chronicle will be reviewed and commented in the following paragraphs.

## MEYNERT'S CONTRIBUTION

Theodor Hermann Meynert (1833-1892) was an outstanding anatomist, neuropathologist, and psychiatrist. He described numerous nervous structures, some for the first time, and also developed theories regarding correlations between neuroanatomical and mental processes.^1-3^

His contribution to the understanding of the basal nucleus is found in his publications where he acknowledges and includes structures previously observed in the upper mesencephalon by Reil and by Gratiolet^4^. It is opportune to write a few words about these two researchers, considering their pioneering descriptions, importance, and Meynert's quotes (Box 1 and Box 2).

**Box 1:** Reil and Die ungenannte Marksubstanz.

Johann Christian Reil (1859-1813), a German anatomist, physiologistand psychiatrist, in the paper *Das Hirnschenkel-System oder die Hirn­schenkel-Organization im Grossen Hirn*, published in 1809, describes(pp 147-171) the cerebral peduncle and the surrounding structures andwrites in a footnote (p 160 − abridged): *"Die ungenannte Marksubstanz*(the unnamed medullary substance) is a medullated formation in con­nection with the anterior rounded extremity of the optic thalami, placedaround the cerebral peduncle, from inwards to outwards, above and par­allel the optic nerves, ending in the external wall of the lateral ventricle.This substance becomes evident when the optic nerves are lifted awayfrom the cerebral peduncle until the corpus geniculatum. Its real organi­zation and function are not clear to me, thus I have designated it, for now,unnamed *(ungenannte),* until I will have it examined in a special way."5
Apparently he never returned to this subject to analyze its structure anddevise an appropriate designation. However, it may be inferred that hemeant the presence, in the region, of white matter formations.
Reil's region was later named *Substantia innominata* (in Latin).
Two hundred years on, the name is still in use, despite incomplete com­prehension of its complex structure and function.

**Box 2:** Gratiolet and the Anse pédonculair.

**Figure 1:**
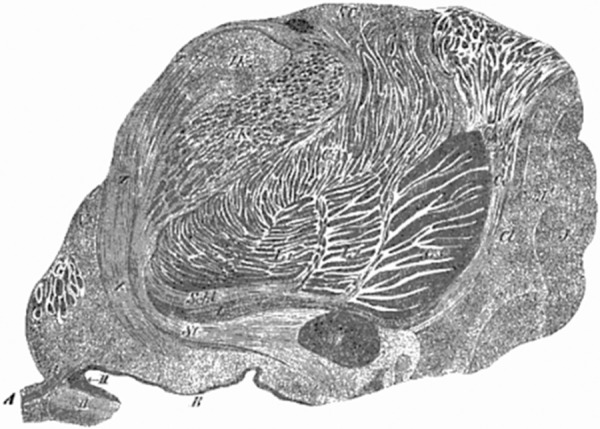
Transverse (coronal) section of the region of the human insula and the basal nuclei (Fig. 245, p 728) (according to Meynert)^4^. *Substantia innomminata or Hirnschenkelschlinge* and its 4 layers: *Schl, L, St, Z* (*Schl*: loop of the lenticular nucleus *[Linsenkernschlinge], L*: ganglion of the cerebral peduncular loop *[Hirnschenkelschlinge], St*: inferior peduncle of the optic thalamus, *Z*: anterior temporal part of the *stratum zonale*). Boundary landmark structures. V: grey matter of the 3rd ventricle; L1, L2, L3: lenticular nucleus; VC: anterior commissure; II: optic nerve; Ce: external capsule.

**Figure 2:**
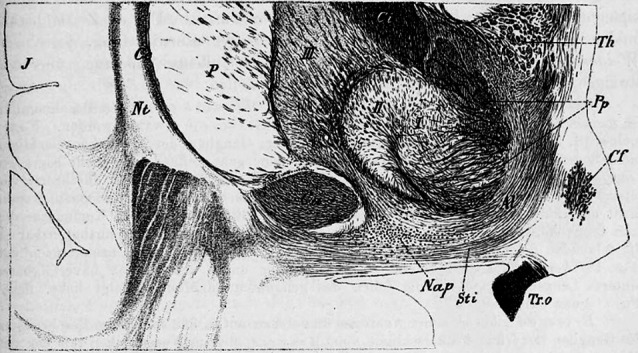
Frontal (coronal) section of human interbrain (Fig. 605, p 457) (according to Koelliker)^10^. Nap: *Nucleus ansa peduncularis* = ganglion of the peduncular loop (*Ganglion der Hirnschenkelschlinge*). Boundary landmark structures: Ca: anterior commissure; lenticular nucleus (I, II, III: Globus pallidus; P: putamen); Ce: external capsule; Tr. o: optic tract; Sti: anterior thalamic peduncle (Stilus anterior thalami); Al: *ansa lenticularis*; Cf: part of *Columna fornicis* (*Säulchen des Gewölbes*) (NB: this Figure is flipped in relation to the former one).

Louis Pierre Gratiolet (1815-1865), a French anatomist, anthropologistand zoologist, in the book published in 1857, regarding the anatomy ofthe cerebral peduncles and beyond (pp 52-72) describes (abridged) an*arcade fibreuse* (fibrous arch), which passes over the peduncular foot,which he named *Anse du pédoncule* (loop of the peduncle),... addingthat it is burrowed along its entire length by a large groove, and "....itsbottom is formed by *fibres blanches mêlées à beaucoup de substancegrise* (white fibers mixed with much grey matter), that house the bandsof the optic nerves, and it will be for us the *goutière de l'anse* (groove ofthe loop)". He also describes "...beyond the *anse,* the concavity of thepavilion pédonculaire*contains a large convex ellipsoid mass which is likethe nucleus (le noyeau)**, whose posterior limit meets the anterior borderof the *Anse pédonculaire* to which it is closely united, and where the sideof the peduncle touches the midline, an extension of this grey matterjoins with a median grey mass that covers the bulge of the intermediaryventricle funnel***- the *tuber cinereum*."6
Gratiolet's mentions the presence of "white fibers mixed with masses ofgrey matter" related to the bottom of the peduncular loop, behind theoptic tract. It is probable that this description was taken by Meynert as a"ganglion" in the course of the *anse.*
He identifies also, a "large convex ellipsoid mass which is like the *nucle­us",* beyond the *anse* (also *corps strié extraventriculaire* [deep part of thebasal ganglia]) (according to Foville). Thus, Gratiolet situates the *anse* atthe transition of the peduncle and the basal ganglia.
The description employs several unusual terms peculiar to the author,and the accompanying illustrations (Plate XXV - Figs. 1-8, explanatorytext pp 38-41) are macroscopic specimens with structures visualized byblunt dissection that are not displayed in a detailed way, hampering anyclear understanding.7

* Upper radiating extension of the peduncular foot or couronne de *l'éventeil pédonculaire* (corona of the peduncular fan) (p 61).

** The nucleus (*le noyeau*) (also *corps strié extraventriculaire*) (according to Foville), probably meaning the deep part of the basal ganglia.

*** Intermediary ventricle funnel: infundibulum.

Meynert published his findings, initially in a chapter of Stricker's book, and later in his own. In Stricker's book (1872 - volume 2 - Chapter XXXI - pp 694-808)^4^ he analyzes the cerebral peduncle and its ganglia (pp 723-734), and describes bundles in the upper mesencephalon, underneath the basal ganglia, that constitute a kind of belting, and emphasizes one which he names *Schlinge des Hirnschenkelfusses* (loop of the peduncular foot), with a course transverse and approximately parallel to the optic tract (Figs. 245 and 247 *Schl*). He relates it to the deepest stratum of the *Substantia innominata* of Reil, or the *Ansa peduncularis* of Gratiolet (p 734). The description is illustrated (Fig. 245, p 728) (Figure 1), and in the legend he designates the structure, describing his findings (p 734) in a synthetic way, as follows: "The *Substantia innominata* of Reil may be divided into 4 layers: (i) the loop of the lenticular nucleus *(Linsenkernschlinge),...(Schl);* (ii) the *Ganglion der Hirnschenkelschlinge* (ganglion of the cerebral peduncular loop) (L),...; (iii) the inferior peduncle of the optic thalamus (St), and (iv) the anterior temporal part of the *stratum zonale* (Z)..."^4,8^

The description of item (ii) above further details the ganglion: "... a flat extended ganglion (Fig. 245 L), that lays below the cerebral peduncular loop, a mass which represents the 2^nd^ stratum of Reil's *Substantia innnominata,* or Gratiolet's *Anse pédonculaire...".* There is also a short description of the cellular component of the ganglion (p 732): "The cells of this ganglion reach the external capsule,...a small number of bundles, isolated or together with others contacting 50 µm long, 15 µm wide fusiform cells..."^4^.

Twelve years later, in his own book (1884 - pp 87-88)^9^, he reiterates his former findings and gives some additional details on anatomical aspects and extent of the ganglion: "...a dense transversely placed particular layer of ganglion cells, parallel to the fiber radiation, which extends laterally until the external capsule...it constitutes a flat well bounded special ganglionic formation, which equals in extension the size of the anterior perforated space"..."this formation shows its cells parallel to the course of the peduncular loop (Ansa peduncularis), being traversed by its bundles, and represents the Ganglion ansae peduncularis (ganglion of the peduncular loop)". He illustrates the anatomical location of the peduncular loop on a finely dissected brain (Fig. 23 ans, p 49). However, there is no depiction or indication of a ganglionic formation (Fig. 27, p 68), despite a well cut section of the region. He did not offer further details or elaboration on the anatomic structure of the region, nor on the cellular structure of this ganglion or its connections, adding very little to his initial description.

## KOELLIKER'S CONTRIBUTION

Albert von Koelliker (born Rudolf Albert Koelliker) (1817-1905), a Swiss biologist, embryologist, histologist and physiologist, in the 6^th^ edition of his book published in 1896 (pp 456-458),^10^, describes in detail the extension, variation in size and position of the ganglion in relation to neighboring structures, as follows: "...The *Substantia innominata* (Reil) *Forel* is the anterior prolongation of the *Zona incerta,* where, appearing as a special formation, beside the already mentioned loop of the lenticular nucleus and the inferior thalamic peduncle, lays the ganglion of the peduncular loop (Fig. 605, p 457) *(Ganglion der Hirnschenkelschlinge [Nap][Nucleus ansa peduncularis])* (Figure 2), as named by Meynert..." . After describing several arcuate structures related to the cerebral peduncle, he declares: "This ganglion, which I will name the basal ganglion of Meynert *(Meynert'sches Basalganglion)...".* This designation has remained as an eponym of this formation to the present day. He follows with a detailed description of the cell clusters of the basal ganglion, analyzing numerous sections, three of them illustrated (figs. 598, 599, and 605), and the changes in size as it spreads out among the bounding structures, the posterior end of the mammillary bodies, underneath the lenticular nucleus and the radiation of the anterior commissure, above the anterior perforated space and the optic tract, medial to the grey matter of the 3^rd^ ventricle wall and to the septal area, the external capsule as the lateral limit, and the anterior limit represented by the region of the floor of the inter-hemispheric fissure, where it gradually terminates.

Regarding the histology of the ganglion, he describes: "About the finer organization of the human *Ganglion basale I* cannot report much. Its cells are 20-30 µm large, multipolar, appearing as two rows, one unstained, and the other strongly pigmented, nearly as those of the *Locus coeruleus.* Around the cells everywhere are found well developed delicately woven fibers, though in the Weigert's preparations I examined there was not any hint about the course of the axons of these cells".

Reviewing the literature, Koelliker affirms that Meynert's ganglion of the peduncular loop, his "basal ganglion", as far as he knows, was mentioned only by Forel, Brissaud, and Honegger. However, no relevant information was added to the knowledge on this subject.^10^

## BEYOND THE PIONEERING STUDIES

After Meynert's and Koelliker's contributions, the latter responsible for the eponym (nucleus basalis of Meynert – nbM), the region was apparently disregarded for a long period. Although additional details of the surroundings of the region were described in the years that followed, several authors made no mention of it at all, possibly due to its unknown relevance^3^. After some time, the initially "unnamed medullary substance" was intensely scrutinized, and numerous studies appeared focusing on the histological segmentation of this richly populated region in several divisions and nuclei, adding other related ones, such as those of the diagonal band and of the septum. The development of specific histological techniques, allowed the identification of cells according to their neurotransmitters, and among them the detection of cholinergic neurons in the late 1970s and early 1980s. A new nomenclature was also proposed to identify the several neuronal clusters of the nbM and associated nuclei, and these were also identified as the major source of long projection neurons for cholinergic innervations^11^. These studies were paralleled by pathological demonstration of neuronal loss in the nbM in brains of patients with Alzheimer, and later in other neurodegenerative diseases. During this same period, functional studies about the relationship between cholinergic deficiency and memory loss were also carried out, which culminated in the proposal of a "cholinergic hypothesis", and later in the development of specific therapeutic strategies^12^.

## COMMENTS

The 19^th^ century was the time when four personalities, engaged in the study of forebrain basal structures, stood out, namely– Reil, Gratiolet, Meynert and Koelliker.

Meynert described the *Schlinge des Hirnschenkelfusses* (the loop of the peduncular foot), and its ganglion *(Ganglion der Hirnschenkelschlinge)* as one of the layers of *Reil's Substantia innominata,* or *Gratiolet's Ansa peduncularis.* Apparently he drew on Reil's poorly defined topography of the area and Gratiolet's limited report of the region to build up his findings by adding a few structural details and a reduced account on the cellular composition of the ganglion, describing as well as systematizing the components found in this transitional region between the upper mesencephalon and basal formations of the forebrain.

Koelliker renamed the ganglion of the *Ansa peduncularis* or the *Schlinge des Hirnschenkelfusses* with the designation *Meynert'sches Basalganglion* (Meynert's basal ganglion), an eponym that endures until the present day, and described the extension, variation in size and position of the basal ganglion in relation to neighboring structures. Most notable in his observation is the revelation of the wide extent of the ganglion and its cell clusters, which spreads out in the basal region of the forebrain.

Both Meynert and Koelliker described the cell composition of the ganglion or nucleus. However, it is possible to note some differences, with a better account by the latter (remembering he was one of the finest histologists of his time), regarding not only the location and extent of the nucleus, but also its cellular component, concerning the size and the shape of the neurons that each found. It is possible that the authors described cells from different regions, or possibly, from different clusters.

The result of these studies was the establishment of the general topography of the region and the description of the extent and some characteristics of the cells found there, mainly of the so-called *"nucleus basalis"* or *"basal nucleus".* However, the picture given was somewhat incomplete. Nevertheless, this was a seed thrown in a productive field, which in the 20^th^ century would reveal an extraordinary development. A large body of studies appeared, increasing the understanding of the basal nucleus and related formations, benefited by advances in histological techniques and equipment and allied to pharmacological investigation, which revealed the great importance of these structures in the activity of the brain, as evidenced in both normal and pathological states.
